# Assessment of Wound Healing Activity of the Aqueous Extracts of *Colutea cilicica* Boiss. & Bal. Fruits and Leaves

**DOI:** 10.1093/ecam/nep190

**Published:** 2011-02-14

**Authors:** Ipek Peşin Süntar, Ufuk Koca, Esra Küpeli Akkol, Demet Yılmazer, Murat Alper

**Affiliations:** ^1^Department of Pharmacognosy, Faculty of Pharmacy, Gazi University, Etiler 06330, Ankara, Turkey; ^2^Department of Pathology, Dışkapı Yıldırım Beyazit Education and Research Hospital, Ankara, Turkey

## Abstract

The fruiting branches of *Colutea cilicica* Boiss. & Bal., along with leaves and fruits, have been used to heal inflammatory wounds at traditional medicine in various parts of Turkey. In order to evaluate the wound healing activity of the plant, aqueous extracts were prepared from the flowering parts and fruits of *Colutea cilicica* Boiss. & Bal. by using 40°C distilled water. Incision wound healing models by using tensiometer on rats and excision wound healing models on mice were employed to assess the activity. Significant wound healing activity was observed when ointment formulation of the aqueous extract at 1% concentration was applied on the mentioned models. The fruit extract treated the groups of animals, showing a 78.1% contraction in wounds, which was close to the contraction value of the reference drug Madecassol (100%). On the other hand, the same extract used on the incision wound model demonstrated a significant increase (42%) in wound tensile strength, compared to the flowering aerial parts. Histopathological examination also supported the results shown in both the incision and excision wound models. The wound healing effect was evaluated and compared with the reference ointment Madecassol. Moreover, zinc and vitamin C levels in the fruit (9480 ± 0 mcg g^−1^; 389 mg g^−1^) and flowering aerial part (6609 ± 1 mcg g^−1^; 404 mg g^−1^) extracts, which might have contributed to the wound healing process, were determined. The experimental data confirmed the traditional usage of *C. cilicica* Bois*s.* & Bal.

## 1. Introduction

The genus *Colutea* comprises about 28 species (Leguminosae), deciduous flowering shrubs native to Southern Europe, North Africa and Southwest Asia. *Colutea cilicica* Boiss. & Bal., known as “bladder senna”, is native to the Mediterranean, and is grown mostly for its attractive yellow flowers and fruits that hold inflated seed pods having papery walls [1]. The fruits of *C. cilicica,* called “patlak kutnu”, have a significant place in Turkish folk medicine; fruits are usually pounded and mixed with soot from copper kettle and then applied on inflammatory wounds [2]. Some healers used decoctions of the dried fruiting branches to bathe and heal, mostly in abscesses and wounds of kids; other healers used ash of the plant to make ointment in vegetable oil [3].

The plant's wound healing process is promoted through several of its constituents, including active principles like flavonoids, triterpenes and alkaloids [4]. Essential trace elements, especially zinc and vitamin C, also influence the process of wound repair. They act as co-factors or co-enzymes in a number of metabolic functions involved in wound healing [5]. Hence, zinc and vitamin C levels of the aqueous extracts prepared from the flowering parts and fruits were also determined.

The aim of the present study was to investigate, in a scientific platform, the *in vivo* wound healing activity of *C. cilicica*, and to elucidate its traditional use. Aqueous extracts of the plant's flowering aerial parts and mature, seeded fruits were prepared and tested on mice and rats for wound healing activity, using *in vivo* circular excision and linear incision wound models, which have been used together for confirmation of the wound healing activity. Additionally, vitamin C and zinc in the extracts were analyzed to explore their relation with wound healing activity.

## 2. Methods

### 2.1. Plant Material

Flowering aerial parts of *C. cilicica* Boiss. & Bal. (syn. *Colutea arborescens*) were collected from the roadsides of Kalecik, Ankara, in June 2008, and mature fruits were collected in August 2008. The plant was authenticated by Serdar Arslan from Gazi University, Department of Biology, Faculty of Science and Art and a voucher specimen (GUE 2620) was deposited with the Herbarium of the Faculty of Pharmacy, Gazi University, Ankara, Turkey.

### 2.2. Preparation of Plant Extracts

The plant materials were shade dried, and the flowering aerial parts were powdered and mature fruits with seeds were minced into small pieces. Each 50 g of powdered or minced aerial parts was macerated in 40°C distilled water for 24 h. After filtration, the extracts were freeze dried, using a (Lyolab) freeze dryer. The yields of the extracts were 20.1% for flowering aerial parts and 24.6% for seeded fruits.

### 2.3. Wound Healing Activity Tests

#### 2.3.1. Animals

Male Sprague-Dawley rats (160–180 g) and Swiss albino mice (20–25 g) were purchased from the animal breeding laboratories of Refik Saydam Central Institute of Health (Ankara, Turkey).

The animals were left for 3 days at room conditions for acclimatization. They were maintained on standard pellet diet and water *ad libitum* throughout the experiment. A minimum of six animals were used in each group [6–8]. The study was permitted by the Institutional Animal Ethics Committee and was performed according to the international rules relating to animal experiments and biodiversity right.

#### 2.3.2. Preparation of Test Samples for Bioassay

Incision and excision wound models were used to evaluate the wound healing activity. For the *in vivo* wound models, test samples were prepared in an ointment base (vehicle) consisting of glycol stearat, 1,2-propylene glycol and liquid paraffin (3 : 6 : 1) in 1% concentration; 0.5 g of the test ointment was applied topically on the wounded site immediately after each wound was created with a surgical blade, as described in Kupeli Akkol et al. [9].

The vehicle group of animals was treated with the ointment base only, whereas the reference drug group was treated with 0.5 g of Madecassol (Bayer, 00001199), which contains 1% extracts of *Centalla asiatica*.

**Linear Incision Wound Model**

All the animals were anaesthetized with 0.15 cc Ketalar, and the hair on the back of the rats were removed using a shaving machine. Two linear-paravertebral incisions 5 cm long were made with a sterile surgical blade through the full thickness of the skin 1.5 cm away from the midline on each side of the vertebral column [10]. The wounds were closed with three surgically interrupted sutures 1 cm apart. All the sutures were non-absorbable, braided, non-capillery and siliconized. The animals were divided into four major groups: those treated with the extracts, the reference drug and the vehicle, and the negative control group. The extracts, the reference drug (Madecassol) and the vehicle were applied topically once in a day throughout 9 days. The negative control group was not treated with any material. All the sutures were removed on the 9th post-wound day. On Day 10 all the animals were killed under anesthesia. One linear paravertebral incised skin of each animal was measured for tensile strength using a tensiometer (Zwick/Roell Z0.5, Germany), and the other incised skin was sent for histopathological examination [7, 11].

**Excision Wound Model**

This model was used to monitor wound contraction and wound closure time. Each group of six animals was anaesthetized with 0.01 cc Ketalar. The hair on the back of the mice was removed by shaving. A circular wound was created on the dorsal interscapular region of each animal by excising the skin with a 5 mm biopsy punch; the wounds were left open [12]. The extracts, reference drug (Madecassol Bayer) and vehicle ointments were applied topically once a day on the wounds till they completely healed. Progressive changes in the wounded areas were monitored every other day using a camera (Fuji, S20 Pro, Japan). The wounded areas were later evaluated using AutoCAD program. Wound contraction was calculated as a percentage of the reduction in wounded area. A specimen sample tissue was isolated from the healed skin of each group of mice for histopathological examination [13].

### 2.4. Histopathology

#### 2.4.1. Histological Study

Sample tissues were fixed in 10% formalin and embedded in paraffin wax. Serial sections (5 *μ*m thickness) of paraffin-embedded tissues were cut. The tissues were stained with haematoxylin and eosin, which were examined by light microscope (Olympus BX51). Ulceration, necrosis and epithelisation were evaluated in the skin tissues. Congestion, edema, PNL, mononuclear cells, fibroblasts and vascularization were also qualitatively evaluated as −, +, ++ and +++.

#### 2.4.2. Statistical Analysis of the Data

The data on percentage wound healing were statistically analyzed using one-way analysis of variance (ANOVA). The values of *P* ≤ .001 were considered statistically significant. Histopathologic data were considered to be non-parametric; therefore, no statistical tests were performed.

#### 2.4.3. Zinc and Vitamin C Analyzes

Analyzes of vitamin C content were conducted using LC-MS Agilent 1100 series according to methods of Keating (1982), Iwase (1992), Ross (1994) and Zeng (2005) [14–17].

Zinc contents were determined using the in-house validated method of ATAL (Ankara Testing and Analyses Laboratory). In order to analyze the zinc content of the extracts, the samples were digested in Anton Paar Multivave 3000 microwave, and measurments were made with a Perkin Elmer Analyst 800 Atomic absorption spectrophotometer.

#### 2.4.4. Phytochemical Screening

The preliminary phytochemical analysis of the aqueous extracts were studied using the following methods and reagents [18]. For each reaction, 100 mg aqueous extract was used. The total alkaloids were searched using Dragendroff's reagent. The presence of flavonoids were analyzed with metalic Mg plus HCl. For the presence of tannins, the dried extract was dissolved in water, with 10% sodium chloride and 1% gelatin solution. Anthraquinones were analyzed with Borntrager's reaction, whereas saponins, with the capacity of producing foam. Coumarins were detected by adding 1 N NaOH and checking for a blue-green color under UV. Triterpens were tested by mixing the extract with chloroform and then treating the warmed mixture with a small volume of concentrated sulfuric acid [19]. Additionally, the aqueous extracts were submitted to thin layer chromatography (TLC) (Kieselgel 60F254, Merck Art. 5554) using two different solvent systems, chloroform : methanol : water (61 : 32 : 7) and ethyl acetate : formic acid : glacial acetic acid : water 100 : 11 : 11 : 26, as the mobile phase [20]. The flavonoid-type components were visualized first under UV-light and then by spraying the TLC plates separately with Vanilin–H_2_SO_4_, Vanilin–HCl, %5 H_2_SO_4_ and Ammonium vapor and incubated at 100°C for 5 min.

## 3. Results

This study was carried out in order to verify the folkloric claims about *C. cilicica* on a scientific platform. Incision, using tensiometer, and circular excision wound models were employed for assessing the *in vivo* wound healing activity of this medicinal plant. One percent concentration ointments prepared from two aqueous extracts, including different parts (flowering aerial parts and seeded fruits) of the plant, were applied on the experimentally created wounds of rats and mice.

### 3.1. Excision and Incision Wound Models

**Figure 1:**
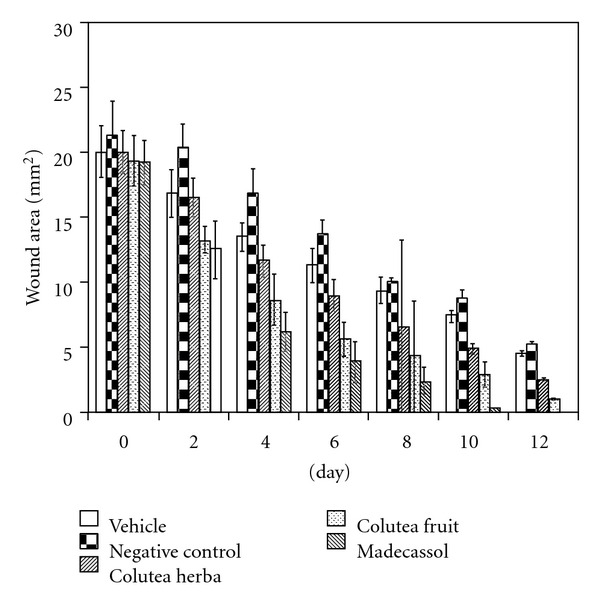
Activities of the extracts from *C. cilicica* on excision wound model.

**Figure 2:**
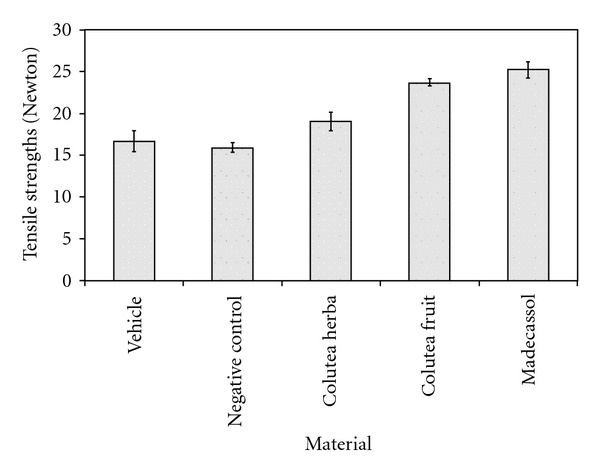
Activities of the extracts from *C. cilicica* on linear incision wound model.

Measurements of the progression of wound healing induced by the aqueous extracts and reference drug on the negative and vehicle groups are shown in Figure 1. Remarkable wound healing activity was observed with the ointment formulation of the aqueous extract of the fruits at 1% concentration on the applied wound models. In the excision wound model, the group of animals treated with fruits extracts showed a 78.1% contraction on Day 12, which was close to the contraction value of the reference drug Madecassol (100%), a widely used plant extract-based pomade. Likewise, the same extract on the incision wound model demonstrated a significant increase in tensile strength on Day 12 (42.0%), compared to the flowering aerial parts (14.30%). The evaluation results of tensile strengths (in Newtons) are shown in Figure 2.

### 3.2. Histopathological Study

**Figure 3:**
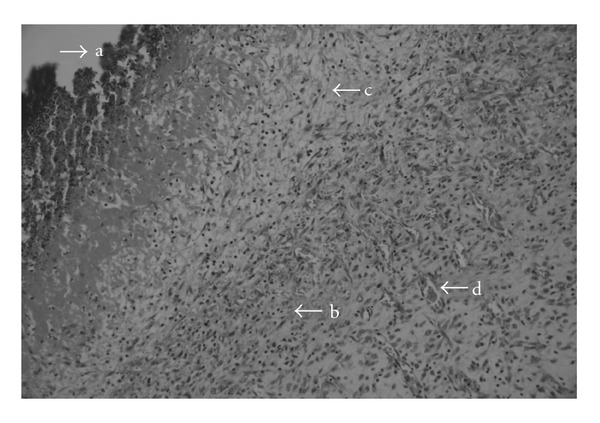
Microscopic view of the section of negative control group (untreated) 10-day-old wound tissue (a) area of ulceration; (b) mixed type inflammatory cells; (c) edema; (d) congested vessel.

**Figure 4:**
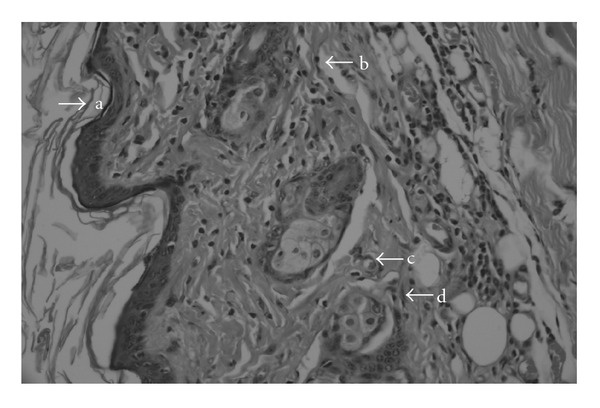
Microscopic view of the section of 10-day-old wound tissue treated with aqueous fruit extract (a) intact epidermis; (b) collagen fibers; (c) blood vessel; (d) fibroblast.

**Figure 5:**
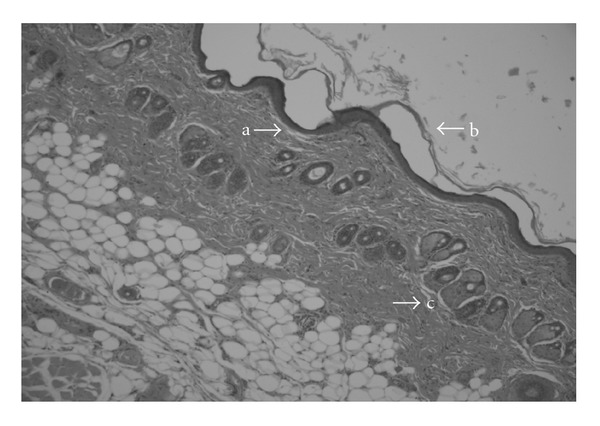
Microscopic view of the section of 10-day-old wound tissue treated with reference material Madecassol (a) intact epidermis; (b) keratinization; (c) hair follicle.

Histopathological examination revealed an obvious ulceration containing fibrin and inflammatory-type cells at the tissue surface of the negative control group (Figure 3). An edema, proliferating vascular structures with congestion, mixed inflammatory infiltration and a plump of fibroblastic cells were observed below the ulceration. On the other hand, as shown in Figure 4, re-epithelization was completed in the wound tissue treated with the aqueous fruits extract, close to the reference drug Madecassol (Figure 5). Moreover, a thin epidermis with keratinization was also observed. Mononuclear inflammatory cells were seen in the dermis, in addition to fibroblasts. Hair follicles existed, and a scar formation was observed in only one of the follicles. A parallel histopathological examination of the reference drug Madecassol demonstrated a complete re-epithelialization with perfect recovery without any ulceration; keratinization of the epidermis, mature dermal layers and hair follicles were observed. A few mononuclear cells and capillary vessels were seen in the dermis. According to results of the studied wound models, re-epitelialization was not observed in the control groups. The results of histopathological examination also supported the outcome of both the incision and excision wound models.

#### 3.2.1. Zinc and Vitamin C Analyzes

The content of the extracts were further evaluated for their zinc and vitamin C levels. Fruit extracts contained 94.80±0 mcg g^−1^ zinc and 3.89 mg g^−1^ vitamin C levels, whereas the extracts of flowering aerial parts contained 66.09 ± 1 mcg g^−1^ zinc and 4.04 mg g^−1^ vitamin C levels, which might have contributed to the wound healing process.

#### 3.2.2. Phytochemical Screening

Preliminary phytochemical screening of aqueous extracts of the fruits and aerial parts showed that both extracts contain flavonoids as major compounds, in addition to moderate levels of tannins, whereas neither alkaloids nor saponosides and triterpenes were detected. Although TLC analyzes of the aqueous extracts of fruits and aerial parts presented a similar profile, there were some bands present in fruits that were not observed in aerial parts. The extracts were submitted to TLC plate with a reference standart flavonol glycoside rutin, which is a general standard used to compare the Rf values and color of unknown bands [20]. Yellow bands without exposure to any reagent indicate flavonol glycosides [21]. When the TLC plate was exposed to ammonia, yellow bands were observed, which became faint brown under UV_366_, most probably indicating flavonol glycosides; non-visible bands after ammonia exposure that under UV_366_ are seen as faint brown most probably indicate isoflavone and flavonones. Moreover, the second TLC plate was exposed to Vanilin-H_2_SO_4_ to observe different components, in addition to flavonoid compounds. Different colored bands were observed under daylight, which are not major components and are yet to be analyzed further. Phytochemical screening of *C. cilicica* Boiss & Ball. is currently under the scope of our team.

## 4. Discussion

In this study, noteworthy wound healing activity was observed with the ointment formulation of the aqueous extract of the *C. cilicica* fruits at 1% concentration on the incision wound model. The natural phases of wound healing include hemostasis, inflammation, proliferation and remodeling. Each step of wound healing is distinct, overlapping the following one and involving a series of interactions between variety of cell classes [22]. Success of wound healing depends on sufficient nutrients being supplied to the wound site; moreover, the objective of wound healing is to heal the wound as quickly as possible, with minimal pain and scarring to the patient [23]. Besides, a flexible and fine scar with high tensile strength is desired for perfect wound closure.

Phytochemical studies on *C. cilicica* revealed that the plant root extract is rich in isoflavonoids [17]. Further, two flavonoid derivatives, coluteol (3′,5′-dihydroxy-7,2′,4′-trimethoxyisoflavan) and colutequinone B (7,4′,6′-trimethoxyisoflavan-2′,5′-quinone), have been isolated from the root bark of *C. cilicica* (common bladder senna) and identified by a combination of ^1^H and ^13^C-NMR techniques [24].

Flavonoids have therapeutic uses due to their anti-inflammatory, antifungal, antioxidant and wound healing properties [25–28]. Moreover, flavonoids and their derivatives are known to decrease lipid peroxidation by improving vascularity and preventing or slowing down the progress of cell necrosis. Hence, any drug that inhibits lipid peroxidation is supposed to increase the viability of collagen fibrils by increasing the circulation and strength of collagen fibers, encouraging the DNA synthesis and preventing cell damage [29, 30]. Flavonoids are also known to endorse wound healing processes primarily owing to their antimicrobial and astringent properties, which appear to be responsible for wound contraction and elevated rate of epithelization [31].

A study on the ethanolic extracts of medicinal and culinary herbs collected from various regions of Turkey showed that aerial parts of *C. cilicica* have *in vitro* antibacterial activity against three Gram-positive (*Bacillus subtilis, Staphylococcus aureus and S. epidermidis*) and two Gram-negative bacteria (*Escherichia coli and Pseudomonas aeruginosa*). Moreover *C. cilicica* (Syn. *C. arborescens*) showed higher inhibitory activity against the yeast *C. albicans* and fungus *A. niger* than the standard antifungal nystatin. The MIC values of the crude extracts of *C. annum, C. arborescens* and *C. cyminum* were found to be between 10 and 17.5 mg mL^−1^ against *E. coli, S. aureus, S. epidermidis, B. subtilis, P. aeruginosa, A. niger, and C. albicans* [23]. In that study, although the aerial parts were not analyzed for their phytochemical content, some antifungal isoflavonoid derivatives might be present in aerial parts as well. Besides antifungal activities of the plant, its fruits have high zinc content compared to other aerial parts.

According to essential trace metal (iron, manganese, cupper and zinc) analyzes conducted on various parts (seeds, fruits, leaves and roots) of 35 selected medicinal and culinary plants, zinc levels mostly varied between 1 and 80 mcg/g, average being 39.1 [32]; zinc contents of a few plant seeds, *Ricinus communis, Mimosa pudica, Cassia absus and Wathania coagulans,* varied between 135 and 498 mcg/g at the same study. The aqueous extracts of fruits and flowering aerial parts of *C. cilicica* were also analyzed using atomic absorption spectrophotometer for their zinc content, and the extract of the fruits demonstrated a much higher zinc content than the extract of flowering parts. Furthermore, the analysis showed that *C. cilicica* aqueous fruit extract had a much higher zinc content than most of the medicinal plants analyzed by Ansari et al. [32].

Zinc is one of the essential trace elements, and serves as a co-factor in various enzyme systems, including zinc-dependent matrix metalloproteinases, which augment autodebridement and keratinocyte migration during wound repair processes. It also provides resistance to epithelial apoptosis via cytoprotection, probably through antioxidant activity of the cysteine-rich metallothioneins, against reactive oxygen species and bacterial toxins. Deficiency of zinc in body, either hereditary or dietary, can lead to delayed wound healing. Oral zinc supplementation might be beneficial in speeding up healing of wounds or treating zinc-deficient leg ulcer patients. On the other hand, studies have shown that topical administration of zinc is superior to oral administration because of its effect in reducing superinfections and necrozis via enhanced local defense systems and the sustained release of zinc ions, which stimulates re-epithelialization of wounds. Zinc is transported through the skin from topical zinc oxide preparations, including paste bandages, that protect and soothe inflamed, ulcerated skin [33]. It is also suggested that traditional herbomineral preparation *Yashada bhasma,* consisting of zinc, has antimicrobial activity. Furthermore, improves the moisture holding capacity of skin, complexion, cell migration and cell regeneration, and thus speeds up the wound healing process [34]. Therefore, zinc content of the extracts used for wound healing purposes might have great contribution in the healing process.

## 5. Conclusion

**Figure 6:**
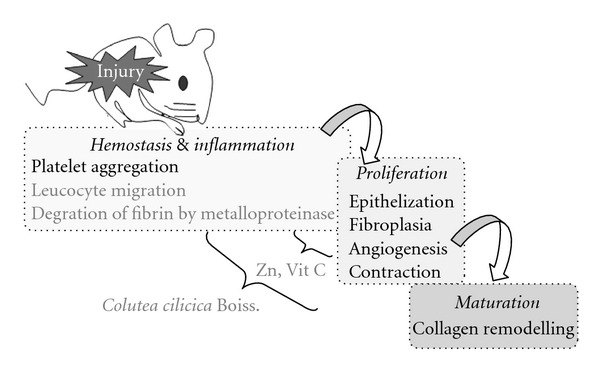
The possible effect of the active extract of *C. cilicica* in wound healing activity.

According to the results reported here, aqueous extract prepared with mature, seeded fruits of *C. cilicica* was found to have better activity on the wound healing experimental models compared to other extract and control groups. Aerial parts of the plant, mostly used as poultice of the fruits, were used in traditional medicine for wound healing. Zinc, vitamin C content and flavonoid derivatives of this medicinal plant might have contributed to the wound healing process, along with other phytochemical contents of the plant. The putative wound healing mechanism of the active extract was presented in the diagram (Figure 6). It seems that the active extract of *Colutea* is effective in both inflammation and proliferation phases. Since Zinc is a co-factor of collagenase, a member of the metalloproteinase group, which assists to remove fibrinogen at the begining of the healing procedure in inflammatory phase [35]. Zinc also contributes in DNA synthesis, cell division and protein synthesis, which occur in the proliferation phase. Vitamin C content of the extract might have a role in enhancing neutrophil migration and lymphocyte transformation along with collagen synthesis. The data represents scientific evidence for the ethnobotanical usage of *C. cilicica* Boiss. & Bal.
